# Supplementary material for: A non-avian dinosaur with a streamlined body exhibits potential adaptations for swimming
Source: Commun Biol. 2022 Dec 1;5:1185. doi: 10.1038/s42003-022-04119-9 (PMC9715538; doi:10.1038/s42003-022-04119-9)
Supplement: Supplementary file 2 — Supplementary Information [file 42003_2022_4119_MOESM2_ESM.pdf]

# A non-avian dinosaur with a streamlined body exhibits potential adaptations for swimming

Sungjin Lee[1], Yuong-Nam Lee[1*], Philip J. Currie[2], Robin Sissons[2], Jin-Young Park[1], Su-Hwan Kim[1], Rinchen Barsbold[3], and Khishigjav Tsogtbaatar[3]

[1]School of Earth and Environmental Sciences, Seoul National University, Seoul, Korea
[2]Department of Biological Sciences, University of Alberta, Alberta, Canada
[3]Institute of Paleontology, Mongolian Academy of Sciences, Ulaanbaatar, Mongolia
*Corresponding author: ynlee@snu.ac.kr

<Contents>

1. Supplementary Note 1
2. Supplementary Figures
3. Supplementary Tables
4. Supplementary References

# 1. Supplementary Note 1

### Additional Description of MPC-D 102/114

### Cranium

The skull is gracile and has an elongated snout (Figs. 1, 2, Supplementary Figs. 1, 2). It is distorted as the preorbital part, especially the left side, is laterally compressed and slightly offset from the orbital region.

The premaxillae are not fused throughout their lengths and display a straight border. As in *Halszkaraptor*[1], but unlike non-halszkaraptorine dromaeosaurids[2–9], the premaxilla is platyrostral, being transversely wider than dorsoventrally high. The premaxillary body is spade-shaped in dorsal view, and its dorsal surface has a shallow depression just anterior to the internarial process. In addition, it bears numerous foramina of various sizes all over its external surface. Extreme pneumatization of the premaxilla is also known in *Halszkaraptor*, representing an extensively developed neurovascular network formed with connected canals[1]. On the anterodorsal surface of the premaxilla, there is an anteroposteriorly developed groove that is bordered by a pair of distinct ridges. The posteriorly bifurcated premaxilla sends two processes that contact the nasal and maxilla, respectively. Dorsally, the internarial process is extremely elongated and overlies the nasal. It posteriorly extends well beyond the level of the anterior end of the antorbital fossa and the posterior margin of the external naris, even though its posterior end is not preserved. It is the opposite of many other dromaeosaurids with short internarial processes. A few dromaeosaurids, such as *Linheraptor*[8] and *Utahraptor*[10], have relatively elongated internarial processes, but they are not long enough to reach the level of the antorbital fossa. Instead, the elongated internarial process of *Natovenator* recalls that of extinct aquatic birds (e.g., *Hesperornis*[11] and *Ichthyornis*[12]). By contrast, the maxillary process is highly reduced, covering less than one-fourth of the ventral margin of the external naris. Between the two processes is a broad, deep subnarial fossa that dominates the posterior part of the premaxilla. As a result of the flat snout, the external naris also faces

dorsolaterally. It is also highly elongated, being about 30% long as the preorbital skull length and extending dorsal to the anterior end of the antorbital fossa. While the long external naris is unusual among dromaeosaurids, it recalls those of some therizinosaurs[13,14]. Posteriorly, the margin of the external naris narrows, giving it a long oval shape. Excluding the subnarial fossa, the anterior margin of the external naris in lateral views is nearly the same level as the ventral margin of the premaxilla-maxilla border, unlike most non-avian theropods. Retracted external nares are also present in *Halszkaraptor*[1], located slightly posterior to the premaxillary oral margin. However, it is unclear how long they are because of the incomplete preservation of the narial region in this taxon[1]. Both maxillae were distorted from crushing but were reasonably intact anterior to the antorbital fenestra.

The maxilla tapers anteriorly and substantially contributes to the ventral margin of the external naris, differing from those of more derived dromaeosaurids[5,8–10,15]. Anterior to the antorbital fossa, the maxilla is very low and elongated as in *Halszkaraptor*[1]. The antorbital fossa is deep, clearly demarcated by thin dorsal and ventral struts. Its ventral part posteriorly becomes a tapering projection that extends to the anterior third of the antorbital fenestra. It also bears an elongated maxillary fenestra which is dorsally positioned. Anterior to the maxillary fenestra is a small promaxillary fenestra. It is small and barely visible in lateral view. The subtriangular antorbital fenestra is longer than high and much larger than the antorbital fossa. Its shape is different from the tall antorbital fenestra in *Halszkaraptor*[1] but similar to the one in many other dromaeosaurids (e.g., *Velociraptor*[3], *Tsaagan*[7], *Deinonychus*[2], and *Austroraptor*[16]), although there are several exceptions such as *Bambiraptor*[4], *Sinornithosaurus*[17], and *Saurornitholestes*[9]. Medially, the maxilla produces a thin floor that marks the anterior margin of the choana. Most of the maxillary alveoli are visible, many preserving in situ teeth. They are subcircular and separated by hourglass-shaped interdental septa connected via the interdental plate. Posteriorly, the jugal process of the maxilla tapers to become a very slender rod.

The nasals are crushed possibly by a bite, given two rows of small openings near the contact with the maxilla. These round apertures are similar in shape and

size and thus could be bite marks of a small predator or scavenger. There is, however, no associated evidence to confirm this speculation. Although the nasals are poorly preserved due to crushing, the right nasal retains most of its anteroposterior extent, nearly as long as the maxilla. Anteriorly, the nasal constitutes more than half of the dorsal margin of the external naris. In contrast, its contribution to the ventral margin is almost non-existent due to the reduced subnarial process. Posterior to the mid-length, the nasal is a long, spine-like bone. No ossified prefrontal bone is preserved in *Natovenator*.

Both lacrimals are preserved but broken and distorted. However, the left one is relatively intact, maintaining its overall morphology. The anterodorsal process has approximately the same length as the posterodorsal process, making the lacrimal triangular in dorsal view. It is different from *Halszkaraptor,* which has a shorter anterodorsal process[1] but more similar to other dromaeosaurids with slender lacrimals such as *Tsaagan*[7] or *Saurornitholestes*[9]. The ventral process is somewhat robust and has a concave posterior surface as well as a lacrimal recess. The preorbital bar appears to be anterodorsally inclined, which is also the case in *Halszkaraptor*[1], although distortion could exaggerate the inclination.

As in other halszkaraptorines[1,18,19], the paired frontals are trapezoidal and arched rather than straight, increasing the relative size of the orbit, and there is also a distinct convexity on the dorsal surface of the posterior half of each frontal. Anteriorly, the frontal conspicuously narrows along the rounded orbital margin. Consequently, the posterior third of the frontal displays a wide lateral expansion, giving rise to the triangular postorbital process near the frontal-parietal suture, which is sinuous. Similar conditions can be found in their frontals in *Halszkaraptor*[1] and *Mahakala*[19]. The part of the orbital rim formed by the frontal is ridge-like and medially produces a shallow groove. The raised supraorbital rim is also known in troodontids[20] and some dromaeosaurids, including *Mahakala*[19]. Inside the right orbit is a remnant of the sclerotic ring, much thinner than other dermal skull bones. There is a limited contribution from the frontal to the supratemporal fossa, evidenced by a small depression on the postorbital process without any clear delimitation. It is

different from the condition in most dromaeosaurids[3,8,9,15,17,21] but very similar to the frontals of *Mahakala*[19] and *Halszkaraptor*[1].

A short anterior portion of the right jugal is preserved, but the contact between the jugal and the maxilla is entirely missing. The preserved part of the jugal is slender and pneumatized without any foramina on the external surface. Medially, it has a concave articular surface that receives the jugal process of the palatine. It also dorsomedially meets the lacrimal, although its border is unclear.

The right postorbital is nearly intact, but the frontal and ventral processes are broken. Overall, it is quite slender like that of *Halszkaraptor*[1]. The postorbital also forms slightly less than half of the lateral margin of the supratemporal fenestra.

$\mu$CT scanning revealed the presence of the left quadrate, which is in articulation with but not fused to the left quadratojugal. The quadratojugal is partially preserved, only retaining a small portion of the posterior region and an isolated bone which is possibly a part of the ascending process. The quadratojugal is generally slender and has a slightly convex lateral surface. On the other hand, the medial surface has a longitudinal groove at the mid-height, where it is the thinnest. Posteriorly, it extends beyond the contact with the quadrate, although its posterior end is missing.

The quadrate is nearly complete but buried in the matrix. According to the $\mu$CT scans, it is gracile and less dense than other cranial elements such as the quadratojugal. It does not have an anterolateral flange, unlike those of some dromaeosaurids[7,17,20]. Instead, the dorsolateral corner of the quadrate laterally projects to make the lateral margin form a wide arc that results in a tall quadrate foramen. The medial margin is also concave, albeit to a lesser extent relative to the lateral margin, giving the quadrate an hourglass shape in posterior view. The quadrate is generally not pneumatized, but a deep and possibly pneumatic fossa dominates the posterior surface of the squamosal ramus, which is posteriorly tilted to meet the concave medial surface of the squamosal. It bears a single robust head that articulates with the squamosal and the lateral wall of the braincase. However, the contact with the squamosal is not limited to the head but extends along most of its dorsal surface, which is inclined

ventrolaterally. Distal to the head, the dorsal articular surface of the quadrate is relatively thin. The pterygoid ramus is thin but greatly enlarged, occupying its base more than half of the quadrate height. It is anteriorly oriented and extensively contacts the quadrate ramus of the pterygoid. Ventral to the two rami, the quadrate shaft gradually expands transversely and produces the articular ramus and quadratojugal processes. The quadratojugal process is pronounced bearing an anterior deflection. Unlike *Mahakala*[19], there is no recess between the quadratojugal process and the pterygoid ramus. The articular ramus is slender and mediolaterally expanded distally. Near the distal end, its anterior surface bears a round fossa. A broader, triangular fossa is also developed on the posterior surface, although it is not as large as that of the squamosal ramus. The distal articular surface of the quadrate is anteroposteriorly short with poorly defined condyles. Medially, the distal surface is dorsomedially inclined to meet the prearticular.

The unfused parietals are incompletely preserved. There is a shallow, transverse concavity right posterior to the frontal and parietal border. The parietal bears a protuberance on its lateral surface, which forms most of the medial wall of the supratemporal fenestra. Posteriorly, a sharp nuchal crest is developed by the parietal and the squamosal, as in other dromaeosaurids[5].

The squamosal is represented only by a string-like bone articulated with the parietal on the right side of the skull. However, the left squamosal is better preserved and articulated with other cranial elements. It is missing its anterior part, which would have met the postorbital and the quadratojugal. The preserved posterior part of the squamosal develops a posterolaterally extending shelf that overhangs the quadrate head, a common character in dromaeosaurids[5,7]. This shelf also posteriorly contacts the paroccipital process below the nuchal crest.

The bones of the posterior braincase, most from the left side, are preserved in articulation (Fig. 2f, Supplementary Fig. 2a–e). The matrix mostly covered them, but it was possible to understand their morphology via $\mu$CT scanning. In general, the preserved braincase bones are incomplete and fused, although a faint border is visible in some cases.

The supraoccipital preserves a small lateral part of its left half that meets the parietal dorsally and the exoccipital laterally. It is anterodorsally inclined, and a deep fossa is anteriorly situated on the external surface. There is no indication of a groove on the preserved part of the supraoccipital, which suggests that the tract for the middle cerebral vein is likely to be located lateral to the midline and dorsoventrally oriented as in *Mahakala*[19].

The exoccipital and the opisthotic are fused into a single element. Laterally, the exoccipital-opisthotic produces a slender paroccipital process that is ventrolaterally oriented like those of *Mahakala*[19] or troodontids[22,23] but unlike the horizontal ones in *Halszkaraptor*[1]. As a result, the paroccipital process extends further ventrally than the level of the occipital condyle. The morphology of the paroccipital process is peculiar, having a broader dorsal surface than the posterior surface, even in its proximal region. In other dromaeosaurids, the paroccipital process is distally twisted so that the pronounced dorsal area is restricted to the distal end[1,7,19,21,24,25]. The laterally notched base of the paroccipital process is nearly vertical and forms the lateral margin of the foramen magnum. Notwithstanding its incompleteness, it is clear that the foramen magnum is greatly widened as in other halszkaraptorines[1,19]. The occipital condyle is missing its dorsolateral part on the right side but retains a round shape. Most of the occipital condyle comprises the basioccipital, whereas the exoccipital only occupies a small dorsolateral part. The occipital condyle appears to be much narrower than the foramen magnum. It is, however, very elongated anteroposteriorly and has an anteroventrally inclined dorsal surface. There is also a lateral constriction or neck at its base, similar to the condition in *Dromaeosaurus* and *Itemirus*[26], as well as some troodontids[23,27].

The lateral wall of the braincase, especially the dorsal region, is poorly preserved. The medial part of the prootic is mostly missing, making it difficult to determine the lateral boundary of the internal chamber for the brain. The prootic heavily contacts the squamosal dorsolaterally, the basisphenoid medially, and the exoccipital-opisthotic posteriorly. Additionally, it has a small ventromedial depression for the quadrate. A wide but rather shallow dorsal tympanic recess is developed on the

lateral wall of the braincase. It differs from the deeply concave ones in other Mongolian dromaeosaurids[7,19,25].

The ventral part of the lateral wall, a fused bone of the basioccipital and basisphenoid, gently rises dorsolaterally, forming a low angle with the braincase floor. A distinct oval opening, possibly connected to the metotic foramen, is accompanied by a depression positioned ventrolateral to the base of the paroccipital process. The braincase floor is also formed by the basioccipital and the basisphenoid. It is missing its anteriormost part and most of its right half. The floor is strongly concave as in *Velociraptor*[25], but its anterior part is less inclined. Its concave middle part is much thicker than the anterior or posterior regions. The anterior half of the preserved floor is perforated by several small foramina, whereas the other half is smooth. The rest of the basicranium is not preserved in the ventral to the occipital condyle and the floor.

The palatal elements are mostly missing or not recognizable except for palatines and the left pterygoid (Fig. 2a–c, Supplementary Figs. 1a, 2a–e). The palatines are partially buried in the matrix and have some broken parts, although the $\mu$CT scanning of the skull could observe their tetraradiate shape. They broadly contact each maxilla and almost meet each other. Besides the poorly preserved pterygoid processes, both palatines are somewhat intact and generally similar in morphology to those of *Deinonychus*[2] or *Velociraptor*[3]. The body of the palatine is thin and dorsomedially inclined but not as steeply as that of *Velociraptor*[3]. As in *Deinonychus*[2] and *Velociraptor*[3], there is a deep recess on the dorsolateral surface of the body that produces the four processes. The choanal process is the thickest among the four. It curves to extend anteromedially and is more elevated than the body. In addition, its anterior articular end is modestly expanded. The maxillary process is markedly elongated, extending anteriorly beyond the middle of the antorbital fenestra. It is unlike the short maxillary processes of other dromaeosaurids[2,3,17]. It is also longer than the choanal process, which is unique among dromaeosaurids, but it is possible that the latter is not entirely preserved. These two processes and the body form the posterior part of the choana, which is considerably elongated considering the position

of its anterior margin. The jugal process is longer than the body and takes part in the lateral margin of the suborbital fenestra. It is also less slender than the maxillary process. The pterygoid process, which only retains its base on both sides, is thinner than the other parts of the palatine. It is a broad, sheet-like bone that directly contacts the choanal process like *Deinonychus*[2].

The left pterygoid is represented by its posterior part, which resembles other dromaeosaurids[2,7,8,17,21], lacking a pronounced pterygoid flange. Laterally, it broadly contacts the quadrate via the sheet-like quadrate ramus dorsoventrally high. The quadrate ramus also produces a small ventral shelf to buttress the pterygoid ramus of the quadrate as in *Dromaeosaurus*[21]. Medial to the quadrate ramus is a deep concavity that is open posteriorly. This fossa is unknown in other dromaeosaurids, and its function is uncertain. It is medially bordered by the shallower articulation surface for the basipterygoid process of the basisphenoid. The articulation surface is quite large and faces posterodorsally, as *Deinonychus*[2] and *Velociraptor*[3]. Furthermore, the basipterygoid notch is also deep, like in *Deinonychus*[2], *Dromaeosaurus*[21], *Velociraptor*[3], and *Linheraptor*[8], although it is relatively broad in *Natovenator*.

**Mandible**

The mandible is slender and similar to that of *Halszkaraptor*[1]. Most of the mandibular elements were observed via $\mu$CT scanning. The dentary is low and elongated, having almost parallel dorsal and ventral margins as in most other dromaeosaurids[1–4,7,9,15–17,21,28,29]. These margins are straight throughout most of their lengths, but the anterodorsal end is slightly raised. A row of small foramina is present near the alveolar margin anteriorly on the lateral surface, and a deep groove is developed posteriorly. A prominent, elongated Meckelian groove is also developed on the medial surface of the dentary, but their anterior extent is unclear because of the heavily damaged medial regions in both dentaries. Unlike some dromaeosaurid taxa[9,29], no anterior concavity is observed on the ventral margin. Posteriorly, the posterodorsal process of the dentary is displaced ventrally from contact with the surangular.

Both splenials are preserved in articulation with each dentary, albeit in poor condition. The splenial anteriorly produces an elongated process that tapers to partially cover the Meckelian groove, posterior to which it forms the majority of the medial surface of the mandible. In lateral view, the preserved parts of both splenials are not exposed. On the right side, only the anterior tip of the surangular is preserved and interfingers with the dentary. The concave ventral margin of this tip suggests that the surangular constituted most, if not all, of the dorsal margin of the external mandibular fenestra.

The posterior part of the left mandible, which includes several postdentary bones, articulates with the quadrate. The left surangular is partially preserved, missing its anterior part. The preserved posterior region is dorsoventrally low but anteroposteriorly long, extending beyond the level of the articulation with the quadrate. It articulates with the angular ventrally and the prearticular medially in the posterior region. Posteriorly, the surangular and the articular are partially fused, which is unusual for a dromaeosaurid. The medial surface of the surangular participates in a large adductor fossa that is also formed by the angular and the prearticular. There is a longitudinal depression on the lateral surface, and a pair of nearly parallel elongated ridges run along the dorsomedial and dorsolateral surfaces, respectively. Together, they define a wide, trough-like shelf that is probably homologous to the prearticular shelf of *Tsaagan*[7] and the shelf for the M. adductor mandibulae externus in *Saurornitholestes*[9]. Other dromaeosaurids are also known to have a dorsally placed ridge or crest on the lateral surface of the surangular[15,17,29]. A small foramen is present below the shelf and anterior to the mandibular glenoid. Another foramen is on the ventromedial surface on the same level as this foramen.

The left angular preserves its posterior part, whose arched lateral half extensively covers the lateral and ventral surfaces of the surangular. In addition, it borders the prearticular along its medial margin. The ventral surface of the mandible is solely constituted by the angular anterior to the mandibular glenoid, at which point the surangular takes its place.

The prearticular is missing its anterior tip. It is low but anteroposteriorly

long with a concave medial surface. Laterally, it is in contact with the surangular, the articular, and to a minimal extent, the medial surface of the distal quadrate. In its preserved part, the prearticular tapers anteriorly, whereas it posteriorly expands to become a major part of the medial wall of the posterior mandible. The posterior end of the prearticular is not well preserved. Notwithstanding, it contacts the retroarticular process while horizontally shifting its orientation. It is, however, not fused to the retroarticular process, unlike *Tsaagan*[7].

The articular is low as other bones of the mandible. The mandibular glenoid, anteriorly bordered by a flat mound, is divided into two subtriangular concavities by a robust ridge. Because of the posterolateral orientation of the ridge, the medial articulation surface is broader than the lateral one. They posteriorly become shallower to form posteroventrally inclined surfaces. The retroarticular process is fan-shaped and dorsally concave. It is thin overall but has a robust medial edge. Posteriorly, it develops a large, dorsomedially oriented protrusion (also called vertical process or vertical columnar process) as in other dromaeosaurids[2,7,9,21]. This protrusion has a concave medial margin and tapers toward its apex.

**Dentition**

The premaxilla incorporates tightly arranged and unserrated teeth that vary in size (Figs. 1a, b, 2a, b, d, e, Supplementary Fig. 1a, c). $\mu$CT scanning revealed 11 teeth behind a broad vacancy in the left premaxilla and 12 teeth on the opposite side. The right premaxillary teeth are followed by a space right anterior to the broken contact with the maxilla. There is also a disarticulated premaxillary tooth that retains its root near the tip of the rostrum. Given that the preserved premaxillary teeth show symmetry in their positions, and the posteriormost left premaxillary tooth is very close to the first maxillary tooth, it is apparent that a total of 13 teeth were initially housed in each premaxilla. Therefore, the number of premaxillary teeth in *Natovenator* exceeds that of any dinosaurs, including *Halszkaraptor* with 11 premaxillary teeth[1]. The premaxillary teeth are differentiated from those in the maxilla or the dentary by having an elongated and incisiviform crown as in *Halszkaraptor*[1].

Further, they have a longer root relative to the other teeth and are not separated by interdental septa in the maxilla and the dentary.

The exact number of the maxillary teeth is not sure due to poor preservation of the posterior parts of both maxillae, although the preserved right maxillary tooth row posteriorly extends to the contact between the maxilla and the jugal. In addition, at least 23 maxillary tooth sockets are recognized on the right side via $\mu$CT scanning. It warrants a higher number of maxillary teeth in *Natovenator* than in most non-halszkaraptorine dromaeosaurids[9], although there are some exceptions, such as unenlagiines with no less than 20 maxillary teeth[15,16]. The maxillary teeth of *Natovenator* are transversely compressed and lack serrations. They are elliptical basally but teardrop-shaped distally with a sharp mesial margin in horizontal cross-sections. The distal carina is, therefore, not easily discerned if present. Compared to the premaxillary teeth, they have a lower and more pointed crown which is strongly recurved. The three anteriormost maxillary teeth on the left maxilla are tiny and closely packed along with the following tooth without segregation, which is similar to the condition in *Halszkaraptor,* where two anteriormost maxillary teeth are much smaller than the following ones as well as the largest premaxillary teeth[30]. Two of these three teeth have broken crowns, but all are clearly much smaller than the more posterior maxillary teeth and most of the premaxillary teeth. Posterior to this cluster, the maxillary teeth become much larger and more loosely spaced with interdental septa that isolate each tooth.

$\mu$CT scanning also revealed the obscured low dentition. Each dentary has about 23–25 alveoli which are generally interrupted by interdental septa. It suggests that the number of dentary teeth is higher than that of typical dromaeosaurids. The dentary teeth are similar to the maxillary teeth in morphology. They are recurved and labiolingually compressed with very weak carinae that do not bear serrations. The crowns of the maxillary and dentary teeth also have a concave distal margin and show uneven heights as in *Halszkaraptor*[1]. Another similarity in the dentition of *Natovenator* and *Halszkaraptor* is that the replacement ofthe premaxillary teeth is prolonged[1]. Therefore, the premaxillary replacement teeth are larger than the small

maxillary ones.

**Axial Skeleton**

The neck is sigmoid due to extreme joint curves, which is unusual for fossil dinosaurs preserved in 3D (Figs. 1, 3a, b, d). Like *Halszkaraptor*[1], it is greatly elongated and consists of cervical vertebrae with long, low centra with a keeled ventral surface. A total of 10 cervical vertebrae are present in *Natovenator*, but most of the 5th cervical vertebra is missing.

The atlas partially preserves the intercentrum and the left neurapophysis, and the former could only be observed by $\mu$CT scanning. The atlas intercentrum is low and anteroposteriorly short. It is solid but not as dense as the neurapophysis. Anteriorly, it has a distinctly concave articulation surface for the occipital condyle. This surface faces anterodorsally to brace the posteroventral surface of the occipital condyle. The dorsal surface of the atlas intercentrum also bears a depression that receives the odontoid process of the axis. Moreover, there is a slight concavity on the ventral surface, which may also be a groove as in other dromaeosaurids[2,7]. Laterally, a short pedicel connects the intercentrum to the neurapophysis. The neurapophysis is thin and missing its dorsal part. It tightly adheres to the axial prezygapophysis, but they are not fused. The contact between them is broad, the concave medial surface of the neurapophysis covering the axial prezygapophysis, and the postzygapophysis is not pronounced. Near the posterior end of the neurapophysis, a thin but dorsoventrally tall projection is on the lateral surface below mid-height. It is posterolaterally oriented and posteriorly twisted. It is a peculiar character not found in other dromaeosaurids.

The axis has a distinct odontoid and a small intercentrum. Otherwise, it is similar in morphology to the following anterior (3rd and 4th) cervical vertebrae. The 6th has the longest centrum and the sharpest ventral keel among the preserved cervicals. The 7th cervical centrum is nearly as long, but from the 8th cervical, the centrum length markedly decreases, as does the distinctiveness of the ventral keel. Accordingly, the three posteriormost cervical centra are stout and transversely less

compressed. All the preserved cervical centra are amphicoelous, as in *Halszkaraptor*[1].

The anterior cervical centra are very low at the anterior articular end but posteriorly become as wide as tall. Unlike many theropods, the cervical centra do not have pneumatic foramina. In other halszkaraptorines, certain cervical centra bear pneumatic foramina or pleurocoels (e.g., the axial centrum of *Mahakala*[19] and the 7th through 9th cervical centra of *Halszkaraptor*[1]). The neural arches of the cervical vertebrae are completely fused to each corresponding centrum, with which they form a large neural canal. In the anterior cervical vertebrae, the posterior extent of the neural arch is on par with that of the centrum like other dromaeosaurids[10]. The neural spines of the axial and 3rd cervical vertebrae are anteroposteriorly long and dorsoventrally low. They are also placed somewhat anteriorly closer to prezygapophyses than to postzygapophyses. Posterior to the 3rd cervical vertebra, the neural spine gradually becomes less distinct and completely disappears posterior to the 7thcervical vertebra. The prezygapophyses are overlapped mainly by the preceding postzygapophyses, so they are not clearly observable in most cervical vertebrae. The only exception is the 3rd cervical vertebra; its left prezygapophysis is well preserved. This prezygapophysis is about a third of the centrum length and laterally diverges slightly. Its medial surface is dorsolaterally inclined and dorsally ends with a sharp ridge. The postzygapophyses on each anterior cervical vertebra are wholly united to make a posteriorly round process without a notched interpostzygapophyseal space, which is a peculiar character also found in *Halszkaraptor*[1]. However, it is uncertain whether this is present on the 5th cervical vertebra like *Halszkaraptor*. There is a pair of ridge-like epipophyses on the axis and posterior cervical vertebrae (7th–10th), unlike *Halszkaraptor* which lacks any epipophyses[1] or other dromaeosaurids with well-developed epipophyses[2,5,7,10,29,31]. The epipophyses anteriorly extend to the mid-length of the centrum but do not exceed the posterior margins of the postzygapophyses. The diapophysis, anteriorly located near the posterior end of the prezygapophysis, faces ventrally in all the preserved cervicals, excluding the atlas. On the other hand, the parapophysis is placed on the anteroventral margin of the centrum. The latter is anteroposteriorly elongated and ventrolaterally directed, making a

subtriangular fossa on the ventral surface of the centrum. As opposed to *Mahakala*[19] or *Halszkaraptor*[1], the diapophyses and parapophyses are not fused with the cervical ribs, which are thin and posteriorly taper.

A total of 12 dorsal vertebrae are observed in *Natovenator* (Figs. 1, 3e, 4a–d, Supplementary Fig. 3a–d). The 11 consecutive dorsals are in a natural position, although the 8th dorsal is poorly preserved. The last dorsal vertebra is separately articulated with the sacrum, which suggests that there could be more than 12 dorsals in *Natovenator*. Only the right side is well exposed in the case of the anterior dorsal vertebrae, and many of the posterior ones are missing neural arches. There is one cervicodorsal vertebra with a keeled and ventrally concave centrum which is longer than the following dorsal centra. The prezygapophyses are obscured, and the postzygapophyses are not much different from those of the posteriormost cervical vertebra. Likewise, the diapophysis is at the same location as those of the cervical vertebrae. The rest of the preserved dorsal vertebrae are similar in length and general morphology. They all have an amphiplatyan or platycoelous centrum that is longer than tall and without pneumatic foramina as in other halszkaraptorines[1,19], although a few posterior dorsal centra are not entirely preserved. The narrow and keeled ventral surfaces of the anterior dorsal centra gradually become wider and flat posteriorly. Unlike others, in the 11th dorsal centrum, the posterior articular surface has a ventral projection. The last dorsal centrum is very low and bears a markedly concave anterior articulation surface. No hypapophysis is observed in the dorsal vertebrae. The parapophyses are on a short pedicel common in other dromaeosaurids[5,6,10,19,28,31–33]. They are located anteriorly and at approximately the same level as the base of transverse processes. It is peculiar among dromaeosaurids because parapophyses of dorsal vertebrae in these dinosaurs are either ventrally placed relative to the transverse processes (e.g., *Deinonychus*[2], *Austroraptor*[16], and *Mahakala*[19]) or horizontal to them only in the posterior dorsal vertebrae (e.g., *Microraptor*[28], *Buitreraptor*[31], and *Velociraptor*[32]). Instead, a similar condition is known in hesperornitheans[11,34,35]. The neural arches of the dorsal series are low, and the neurocentral sutures are not visible. In the anterior dorsal vertebrae, small infradiapophyseal fossae are present.

Their presence in the posterior dorsal vertebrae cannot be known, but it is clear that they become gradually reduced posteriorly and completely disappear in the last dorsal vertebra. Zygapophyses are either missing or indiscernible except in the last dorsal vertebra, which has very short pre-and postzygapophyses. The prezygapophyses are anterodorsally oriented with a low angle with the centrum and face dorsomedially, whereas the postzygapophyses face ventrolaterally. The transverse processes are preserved in the anterior dorsal vertebrae and the last. They are short and narrow in the anterior ones but have a wide base in the latter. Only the 11th and the last dorsal vertebrae preserve a recognizable neural spine. In the former, the neural spine is rectangular with a great anteroposterior length and taller than the centrum. The neural spine of the last dorsal vertebra is damaged but retains its base that appears to be elongated. On the 2nd–7th dorsal vertebrae, ribs are partially preserved on the right side in articulation with each vertebra. The long, slender capitulum is pronounced in contrast to the tuberculum, which is indistinct and nearly fused to the transverse process. Distally, the rib shafts are thin and flat with a rectangular cross-section. Their proximal parts do not extend much ventrally but are horizontal and directed posterolaterally, making a broad arc. It indicates that *Natovenator* had a transversely expanded and dorsoventrally compressed ribcage. The dorsal ribs are also posteriorly oriented to a great extent, forming an angle of 34–48° with each dorsal centrum in ventral view (Table 1). This extreme posterior extension of the dorsal ribs must have streamlined the body as in extant diving birds[36,37].

Several sacral vertebrae and the last dorsal vertebra are preserved in articulation (Supplementary Fig. 3a–d). It is impossible to determine the sacral count as the preserved synsacrum includes the anterior three or four vertebrae. However, the location of the acetabulum indicates that the synsacrum consisted of no less than six vertebrae. The sacral centra are poorly preserved except for the first one. It is fused to the second sacral centrum but not to the last dorsal centrum. It is also slightly wider than long and lacks pneumatic foramina. Its ventral surface is flat without any notable features. The height of the centrum is low, being

approximately the same as that of the last dorsal centrum. There is no infradiapophyseal fossa, and the neural arch is fused to the centrum without open neurocentral sutures. The prezygapophyses are slightly longer and more upturned than those of the last dorsal vertebra. The sacral rib, which is only preserved on the left side, is a thin, broad lamina that is posteriorly located. Like most other dromaeosaurids[5], the neural spines are fused into one elongate plate. It is roughly four times as tall as the sacral centra. Only a small part of the second sacral centrum is preserved, and it is unclear whether it bears a groove on its ventral surface. The third and possibly fourth sacral vertebrae are primarily represented by the fused neural spine and neural arch.

The caudal series is incomplete and partially articulated (Fig. 3f, Supplementary Fig. 3e–i). In general, the morphology of the caudal vertebrae is very similar to that of other halszkaraptorines[1,19]. The preserved caudal vertebrae consist of three separated sets, and several vertebrae are in articulation within each set. Within each set, centrum lengths vary and do not show any lengthening or shortening tendency. In general, however, it appears that posterior caudal centra are longer than anterior ones as the members of the posteriormost set are the longest. The first set includes five consecutive caudal vertebrae, which are strongly curved. They are most likely from the anterior region of the tail, judging from dorsoventrally high centra, pronounced transverse processes, and large chevrons. Even though it is not possible to determine the exact position of the first set in the caudal series, this set exhibits morphology most similar to that of the 2nd–6th caudal vertebrae of *Mahakala*[19]. The second set has four vertebrae that are positioned above the sacrum. Their position, low centra, and ridge-like transverse processes indicate that they were posteriorly positioned relative to the caudal vertebrae from the first set, and the transition point is likely to have lain between these sets. The third set, comprised of six vertebrae, is considered the most posterior among the preserved sets based on lower and narrower centra, absence of transverse processes, and greatly reduced zygapophyses. The caudal vertebrae of the first set have a platycoelous centrum which is relatively elongated like those of other halszkaraptorines[1,19]. The anterior

articular surface is wide and subrectangular. On the other hand, the posterior articular surface is nearly square-shaped. The centra in the other sets are lower and narrower, and several from the third set are transversely compressed. Besides the anteriormost one, the caudal centra of the third set develop a pair of fossae on each lateral side. They are small pits in more anterior centra but posteriorly become much larger and elongated. Two distinct ridges bound the enlarged fossa; a similar structure is also known in *Buitreraptor*[31,33,38] among dromaeosaurids. There are no pneumatic foramina on any of the preserved caudal centra. Most of them have an anteroposteriorly elongated lateral ridge slightly above the mid-height. They also have a deep sulcus on the ventral surface, which runs through the entire centrum length. None of the prezygapophyses has extreme elongation in many other dromaeosaurids[2,28,32,39–43]. The prezygapophyses of the anterior two vertebrae from the first set are large, almost horizontal, and anterolaterally extended. These features are shared by both *Halszkaraptor*[1] and *Mahakala*[19] in their anterior caudal vertebrae but not with many other dromaeosaurids[5,28,31,33]. In contrast to these two vertebrae, the following three have more inclined prezygapophyses whose facets face dorsomedially. As for the second set, the zygapophyses are mostly broken off or obscured except for the prezygapophyses of the third vertebra. These prezygapophyses are still quite large and more vertical. In the third set, the prezygapophyses become slenderer but remain pronounced. The postzygapophyses of the anterior caudal vertebrae are horizontal and raised very little, as in *Mahakala*[19]. Unlike this taxon, however, they posteriorly extend beyond the level of the centrum, albeit very slightly. The postzygapophyses are dorsally tilted in more distal caudal vertebrae and laterally diverge. The neural spine is poorly preserved in most of the preserved caudal vertebrae. In the first set, the two anteriormost vertebrae had a broad base for the neural spine, whereas the base is transversely thin in the following vertebra. Additionally, the neural spine of these three vertebrae is restricted to the posterior part as in the anterior caudal vertebrae of *Mahakala*[19]. Several incompletely preserved neural spines in other sets are low and elongated ridges. The transverse processes of the anterior caudal vertebrae in the first set are thin and have an abroad base, but

their orientation is unclear. As stated for *Halszkaraptor*[1], the two anteriormost vertebrae have a distinct zygodiapophyseal lamina on each side. Caudal vertebrae have greatly reduced transverse processes that posteriorly become more inconspicuous than the first set.

The chevrons are preserved in articulation with the caudal vertebrae of the first and third sets (Figs. 1c, d, 3f, Supplementary Fig. 4e, h, i). In the former, the two preserved chevrons are as large and broad as the anterior chevrons of other dromaeosaurids such as *Halszkaraptor*[1] and *Velociraptor*[32]. The anterior chevron is much larger than the posterior one and dorsoventrally twice as high as the articulating centrum. The chevrons in the third set are smaller and anteroposteriorly bifurcated to form an inverted T shape with a longer and slenderer anterior process. Their ventral surface is transversely narrow, as in *Mahakala*[19]. It also lacks a sulcus or overlapping, unlike *Buitreraptor*[31,33].

**Appendicular Skeleton**

The pectoral girdle of *Natovenator* includes a right scapula located on top of the dorsal ribs (Supplementary Fig. 2), fragmentary coracoids (Fig. 1a, b), and a furcula (Fig. 1a, b).

The scapula only preserves a partial scapular blade, which is narrow and laterally bowed. It appears to be in a natural position but is missing both ends, so much anatomical information cannot be gathered.

Two thin and rectangular bones, articulated with each of the clavicular rami of the furcula, are interpreted here to be a part of the left and right coracoids, respectively. Whether the coracoid was fused to the scapula in life is not certain. Without any distinct character, they are flat with a worn surface. The left coracoid is associated with a long, straight, rod-like bone, which is possibly a sternal rib or a gastralium.

A slender furcula is completely preserved except for the broken epicleideal regions. It is boomerang-shaped and has an interclavicular angle of approximately 115°, which is relatively wide compared to those of many other dromaeosaurids such

as *Velociraptor*[5,32], *Buitreraptor*[31,33], *Bambiraptor*[41], *Tianyuraptor*[44], *Changyuraptor*[43], and *Microraptor*[28] but narrower than that of *Zhongjianosaurus*[45]. The two clavicular rami are nearly symmetrical and dorsally taper. The furcula also lacks a hypocleidium as in *Tianyuraptor*[44], *Bambiraptor*[41], and *Microraptor*[28]. Its cross-section is subtriangular due to a slightly pointed posterior surface.

Both humeri are preserved in *Natovenator*, but only the right one is well exposed for examination (Fig. 2a–d). The right humerus is located above the scapula. Its precise length is not certain because of the poorly preserved extremities. The preserved humeral length (about 86% of the preserved length of the femur, which is estimated to be 60–70% complete) suggests that the humerus was relatively short compared to those of many paravians but similar to ones in other halszkaraptorines[1,19]. The proximal region of the humerus is strongly deflected in the posteromedial direction and bears a triangular concavity on the medial surface. The humeral head and internal tuberosity are poorly preserved, the former being represented only by a small bump. Similarly, the deltopectoral crest is broken off, excluding its distal end. However, the base of the deltopectoral crest is anteriorly oriented and confined to the proximal third as in other dromaeosaurids[2,5,28,31,33,41,44,45]. The humeral shaft is twisted, so its proximodistal axis is sigmoid, like in some unenlagiines[31,46] and *Deinonychus*[2]. Distally, the anteromedial surface of the shaft becomes flat and wide, forming a concavity, whereas the posterolateral surface is convex. The distal articular ends are mostly missing, but the anterolaterally tapering broken distal surface indicates the more robust ulnar condyle than the radial one. The distal end also anteriorly curves to make a depression on the anteromedial surface, possibly shared with the *Austroraptor*[31,46].

Only the left ulna is preserved with its medial side exposed. It is missing its proximal portion, and its distal end is also heavily worn. Despite its incompleteness, the ulna is estimated to be a little shorter than the humerus based on the in situ layout of the entire left forelimb, presumably representing a natural articulation. The nearly straight shaft anteroposteriorly expands toward the distal end. Additionally, it is mediolaterally compressed to a great extent, the posterior surface

being particularly narrow with a sharp margin as in other halszkaraptorines[1,19].

The left radius is preserved, but only its distal half is visible. It is slenderer than the ulna, whose shaft is twice wider anteroposteriorly at the estimated mid-shaft regions. The anteroposterior width of the preserved part of the radius is virtually consistent, whereas the mediolateral width distally increases. As with the ulna, the distal articular surface of the radius is eroded.

Two carpal bones are partially preserved in articulation with the left ulna and radius as well as the left manus. The more distally located element in contact with the metacarpals is interpreted as semilunate, and the other may be the ulnare (or 'scapholunare' in Botelho et al.[47]). However, little can be said about their morphology due to poor preservation. The preserved portion of the semilunate is semicircular and covers the entire proximal surface of metacarpal II, although it barely meets metacarpal I.

The left manus is articulated with the antebrachium and folded, with almost a right angle between them. It is slender and much elongated, as suggested by the second manual digit. The preserved left manual elements comprise three metacarpals and several manual phalanges, most of which can only be observable in ventral view.

Metacarpal I is stout and the shortest among the metacarpals, less than half the length of the others. Its proximal end is obscured and seems to be quite damaged. The shaft of metacarpal I has a straight lateral margin and slightly concave medial margin, which is similar to *Buitreraptor*[33]. Distally, there is a small flex or pit just proximal to the lateral condyle on the ventral surface, otherwise flat. The distal articular end of metacarpal I is ginglymoid, and it has small medial and lateral condyles well separated by a wide groove. The lateral one is much larger between these condyles and extends more distally than the medial one.

Metacarpal II is shorter than metacarpal III, which is unusual among theropods. Its proximal third is robust and has parallel medial and lateral margins in ventral view. Distally, however, the medial and lateral surfaces of metacarpal II have a wide concavity giving it an hourglass shape. The ventral surface of the proximal half is

damaged, whereas that of the distal half is intact and gently concave. The distal articular end of metacarpal II appears to be ginglymoid, but its structure is not visible due to erosion.

Metacarpal III is mediolaterally as wide as that of metacarpal II at the mid-shaft as in other halszkaraptorines[1,19]. It is also slightly twisted, with its proximal third being ventrally offset from metacarpal II. The ventral distal part of the medial surfaces is concave, and the mediolateral width of the former distally increases. Both proximal and distal articular ends are poorly preserved.

A total of five left manual phalanges are preserved in partial articulation, but none of them are ungual phalanges. Phalanx I-1 is the only phalanx preserved in the manual digit I and articulated with metacarpal I. It is relatively longer than that of *Halszkaraptor*[1], being more than half the length of metacarpal II. Still, it is short compared to those of other dromaeosaurids such as *Deinonychus*[2] or *Buitreraptor*[33]. Proximally, a prominent longitudinal groove halves the ventral surface but does not much extend distally. Although only the lateral half of the distal articular end is exposed, it is ginglymoid, exhibiting a pronounced condyle that proximally produces a low and short ridge.

Two proximal phalanges of the second manual digit are preserved in articulation. Manual phalanx II-1 is nearly half the length of metacarpal II. Its ventral surface is fractured and covered by a matrix, but its hourglass-shaped outline is recognizable.

Manual phalanx II-2 is missing its distal end, but it is still longer than phalanx II-1. It is straight and distally tapers until it mediolaterally widens near the distinctly developed flexor pit. Moreover, it is much wider than tall and has a soft concave ventral surface.

The two preserved phalanges of the third manual digit are not in articulation but are closely associated with metacarpal III. It is, therefore, not possible to identify their original position. Although it is uncertain whether all the phalanges in *Natovenator* had similar lengths as in *Halszkaraptor*[1], they are subequal in length, unlike those of many paravians, including dromaeosaurids[10,33]. One of the two phalanges is only

visible through its longitudinal cross-section. In contrast, the broken lateral surface and a small part of the ventral surface are exposed to the other. In general, the former is dorsoventrally higher than the latter. The latter manual phalanx has a reasonably deep ligament pit on the lateral distal condyle.

The pelvic girdle of *Natovenator* includes both ilia and pubes, all of which are only partially preserved (Supplementary Fig. 3a–d). The ilia, articulated with the sacrum, are represented mainly by their middle regions and inclined towards the sacral neural spine. A well-developed lateral ridge is present on the preacetabular blade, separating the cuppedicus fossa from the lateral surface of the ilium and forming an antiliac shelf as in other dromaeosaurids[28,32,33] but unlike *Mahakala* that does not have an antiliac shelf[19]. Each ilium has a concave lateral surface bearing a small acetabulum and lacks a supracetabular crest. The pubic and ischial peduncles are missing in both ilia.

The pubes retain only their proximal parts and are retroverted, and they are in close articulation with the ilia and do not seem to deviate far from their natural positions. In addition, they transversely widen toward the proximal end, probably reflecting that a robust pubic peduncle was present on the ilium.

The hind limb elements of *Natovenator* consist of a left femur, a left tibia with an astragalocalcaneum, and a right pes, none of which are complete (Figs. 1a, b, 3g, 4a, Supplementary Figs. 3a–d, 4). The left femur preserves only its diaphysis with a severely crushed distal part and is missing both proximal and distal ends. Additionally, it is positioned so that only its posterior side is visible. The femoral shaft is anteriorly bowed to a slight extent. A low and elongate ridge is developed along most of the preserved posterior surface near the posterolateral margin. This ridge, which may represent a linea intermuscularis caudalis[48], could be homologous to the crest that is continuous with the ectocondylar tuber in other halszkaraptorines[1,19] and possibly *Rahonavis*[49].

The left tibia is partially preserved, retaining only its distal part and tightly articulated with the astragalocalcaneum (Supplementary Fig. 4a–d). It has an elliptical cross-section for most of its length, and its mediolateral width increases

distally. The anterior and posterior surfaces are mainly flat except for a couple of concavities near the distal end on each side, likely resulting from postmortem crushing. A small bump is developed on the posterolateral edge of the distal end and bears a low crest. The two malleoli are nearly on the same plane, the lateral one being only marginally extending further distally than the medial one.

The astragalus and calcaneum are fused in other dromaeosaurids except for *Deinonychus*[2]. They are, however, not fused to the tibia, and the lateral and medial extremities of the astragalus are partially broken. The ascending process of the astragalus only covers the lateral half of the anterior surface of the distal tibia, although this is likely to be a result of breakage. Its proximal end is also missing, but the distinctly marked surface of the tibial shaft, which appears to be the articular surface for the ascending process, indicates that this process was proximodistally elongated with a tapering proximal end. A shallow transverse groove separates the ascending process from the astragalar body. The two condyles are well defined and intersected by a mediolaterally wide intercondylar groove. Although they are not entirely preserved, the medial condyle is larger than the lateral condyle. Additionally, the anterior extent of the medial condyle exceeds that of the lateral condyle, whereas the reverse is true for their distal extent. Most of the proximal part of the calcaneum is not preserved. Thus it is not certain whether there was a facet for the fibula. Distally, a slight concavity is present on the lateral surface of the calcaneum. The proximal tarsals do not invade the posterior surface of the tibia, unlike those of *Mahakala*[19].

The right pedal elements of *Natovenator* are mostly in articulation with each other (Fig. 3h, Supplementary Fig. 4f–h). They include metatarsals II–V, an ungual phalanx of pedal digit I, pedal phalanges II-1 and III-1, and the entire pedal digit IV. Only the shaft of metatarsal IV, metatarsal V, and the pedal ungual phalanx of the first digit are separated but very closely associated with other elements.

Metatarsals II and III are mostly missing, excluding their distal segments. However, it is possible to deduce from the articulated state of the right pes that metatarsal II is proximodistally shorter than metatarsals III and IV with their distal

extents. Moreover, metatarsal II is slenderer than metatarsal III, especially in ventral view. The broken surface of the shaft of metatarsal II reveals a sub-rectangular cross-section. Distally, metatarsal II medially deflects away from metatarsal III, like in *Mahakala*[19] or *Rahonavis*[49]. The distal end of metatarsal II has a ginglymoid trochlea as in most other dromaeosaurids[5,10]. A wide, deep intertrochlear sulcus separates the asymmetrical two condyles. These condyles do not have a ligament pit. The lateral condyle is more robust than the medial condyle, but they are much slenderer than the condyles of metatarsal III.

Distally extending further than other adjacent metatarsals, metatarsal III would be the longest among metatarsals if it reaches the proximal articular end. It does not have a mediolaterally expanded dorsal surface that overlaps metatarsal II nor bear a constriction near the distal end, which is different from the conditions in *Mahakala*[19] or *Hulsanpes*[50,51] but shared with *Halszkaraptor*[1] among halszkaraptorines. However, the distal shaft is not completely preserved. The dorsal surface of the distal shaft of metatarsal III is mediolaterally wider than the ventral surface due to the slanting lateral surface that produces a pair of proximodistally oriented ridges. The more ventrally located ridge probably marks the dorsal limit of the contact with metatarsal IV and disappears near the distal end. Ventrally, a third ridge marks the border between the lateral and ventral surfaces. Proximal to the third ridge, the ventral surface is not well defined but rather confluent with the lateral surface. As a result, the ventral surface proximally tapers to nearly become pinched by metatarsals II and IV, unlike other halszkaraptorines[1,19,50,51]. Nevertheless, metatarsal III of *Natovenator* is too incomplete to determine whether the metatarsus has a subarctometatarsalian condition comparable to that of Jehol dromaeosaurids[28,40,42,44,45] or unenlagiines[31,33,46,52]. Metatarsal III also has a ginglymoid distal articular surface like metatarsal II, but its intertrochlear sulcus is not wide. The two distal condyles of metatarsal III are almost identical in mediolateral width. However, the medial condyle is proximodistally longer and proximally extends further than the lateral condyle in ventral view. No ligament pits are visible on both condyles.

Metatarsal IV preserves a partial shaft and distal articular end, and the

former is not connected to the latter but is closely associated. Proximally, the shaft of metatarsal IV is dorsally expanded and subtriangular in cross-section with a sharply ridged dorsal surface. Although the proximal articular end of metatarsal IV is missing, the proximal articular surface was also subtriangular and taller than wide. The cross-section distally becomes elliptical as the dorsal ridge diminishes. Another ridge, which contacts metatarsal V, is developed on the lateroventral surface near the proximal end. The distal end of metatarsal IV is badly damaged but has a single condyle with a small lateral flange. It also maintains its contact with metatarsal III and does not diverge as opposed to that of *Hulsanpes*[50,51]. Metatarsal V is extremely reduced and slightly curved. The morphology of metatarsal V is quite simple, being very slender except for the relatively robust mid-shaft.

The first pedal ungual phalanx is disarticulated from other pedal elements but closely positioned to phalanx II-1. It is almost complete, only missing its distal tip. Further, it is mediolaterally narrow, moderately curved, and very similar to the ungual phalanx of the fourth digit in size like that of *Mahakala*[19]. A small proximodorsal lip overhangs the mediolaterally narrow proximal articular surface, below which a pronounced flexor tubercle is present, albeit partially broken. There is a groove that distally becomes more distinct on each side, as noted in *Rahonavis*[49]. The grooves, however, are not symmetrical regarding their depth and position. Like *Buitreraptor*[33], the lateral groove is deeper and more dorsally located than the medial one that divides the distal part of the ungual in half. Pedal phalanx II-1 is much shorter than phalanx IV-1, which differs from the condition in *Buitreraptor*[33] with phalanx II-1 similar to phalanx IV-1 in length. Notwithstanding, pedal phalanx II-1 of *Natovenator* is relatively stout, maintaining its mediolateral width along the entire length. The proximal end of phalanx II-1 is broken but retains the proximodorsal end with a flat proximal margin. There is a deep, circular extensor pit on the dorsal surface near the distal end, which is ginglymoid with a proximodorsally extensive intertrochlear sulcus for hyperextension of phalanx II-2, as is commonly known in other dromaeosaurids[5,31]. The distal condyles are large and nearly symmetrical. On the other hand, the lateral ligament pit is much shallower and less defined than the

medial one.

PhalanxIII-1 is missing its distal part and is poorly preserved in general. The medial surface is crushed, and the proximal region of the ventral surface is heavily worn. Still, it is the most robust among the preserved phalanges. The phalanges of the fourth digit are complete and almost intact. Phalanx IV-1 is straight and elongated, being proximodistally much longer than phalanx IV-2, unlike *Mahakala*[19], which has a short and medially bowed pedal phalanx IV-1. Additional differences between them include the proximal shaft with a lack of a dorsoventral constriction (present in *Mahakala*[19]), the presence of a pair of ridges on the ventral surface of the proximal end (absent in *Mahakala*[19]), and the deeper medial ligament pit than the lateral one (reversed in *Mahakala*[19]) in *Natovenator*. Nonetheless, the narrow shaft with a subquadrangular cross-section is similar to that of *Mahakala*[19]. A pronounced dorsoventral constriction is formed proximal to the distal articular end by a distally tapering shaft and the expansion of the distal end. The extensor fossa is large and deep compared to the small and shallow flexor pit. Compared to phalanx II-1, the ginglymus of phalanx IV-1 is less pronounced and does not extend proximally. The distal condyles are symmetrical, although the medial ligament pit is much larger than the lateral one. In the more distal phalanges of the same digit, the medial ligament pit is deeper than the lateral one, whereas they are similar in size.

Phalanx IV-2 is short but has a wider proximal end than phalanx IV-1. Its dorsal side is obscured and not visible. Proximally, the ventral surface expands into a round and slightly concave plane with a sharp margin. It is distally followed by a narrow shaft and wider distal end, both of which contribute to the formation of a mediolateral constriction of the ventral surface slightly proximal to the mid-shaft. Distal to the constriction, the flexor pit on the ventral surface is deeper than that of phalanx IV-1. The distal articular end is ginglymoid and has a pair of ventrally parallel condyles, unlike *Mahakala*[19] with a ventral divergence. However, the medial condyle is wider than the lateral one in ventral view, as in *Mahakala*[19].

Phalanx IV-3 is shorter but slightly different from the preceding phalanx, with a few differences. On the ventral surface of the proximal end, the concavity present

in phalanx IV-2 is smaller and bordered by a pair of wide ridges on each side. In addition, the mediolateral constriction is positioned more distally and close to the mid-length, and the lateral distal condyle extends further distally than the medial one. The dorsal surface is obscured like that of phalanx IV-2.

Phalanx IV-4 is noticeably shorter than phalanx IV-3, which differs from *Mahakala*[19] or *Buitreraptor*[33], where they are subequal in length. This phalanx is also mediolaterally narrower than phalanx IV-3. At the proximal end, there is a well-developed proximodorsal lip, and the flat ventral surface has a small proximal protrusion in the middle as well. The mediolateral constriction is less prominent in comparison to the more proximal phalanges. There is a small extensor pit on the dorsal surface, and the flexor pit on the ventral surface is negligible. The distal trochlea is ginglymoid but more reduced than the other pedal phalanges. The ligament pits are relatively large and dorsally located.

The ungual phalanx of the fourth pedal digit is not as curved or mediolaterally compressed as the first pedal ungual. The proximodorsal lip is very elongated, but the flexor tubercle is much smaller than the one in the ungual of the first digit. Distal to the flexor tubercle, the ventral surface is flat and distally tapers. Unlike the ungual of the first digit, the grooves on the lateral and medial surfaces are well defined throughout their lengths. Their ventral extension is very pronounced, almost contacting each other on the flexor tubercle.

## 2. Supplementary Figures

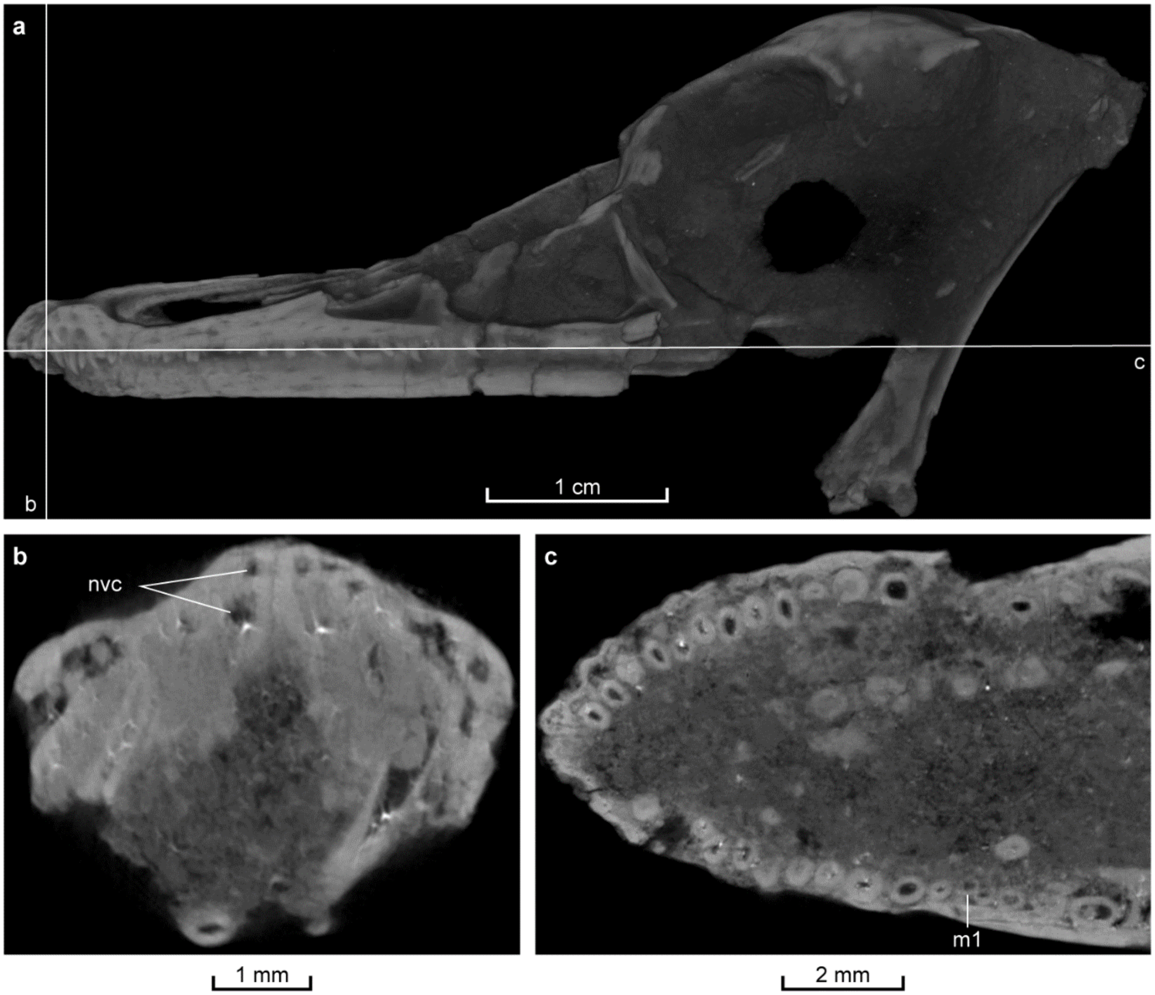


**Supplementary Figure 1.** $\mu$**CT rendered images of the skull of *Natovenator polydontus* (MPC-D 102/114, holotype).** **a**, Skull in left lateral view. **b**, Slice of the snout region marked on **a** showing the neurovascular chambers in anterior view. **c**, Slice of the upper dentition marked on **a** showing the cross-section of the premaxillary and anterior maxillary teeth in dorsal view. Abbreviations: nvc, neurovascular chamber; m1, 1st maxillary tooth.

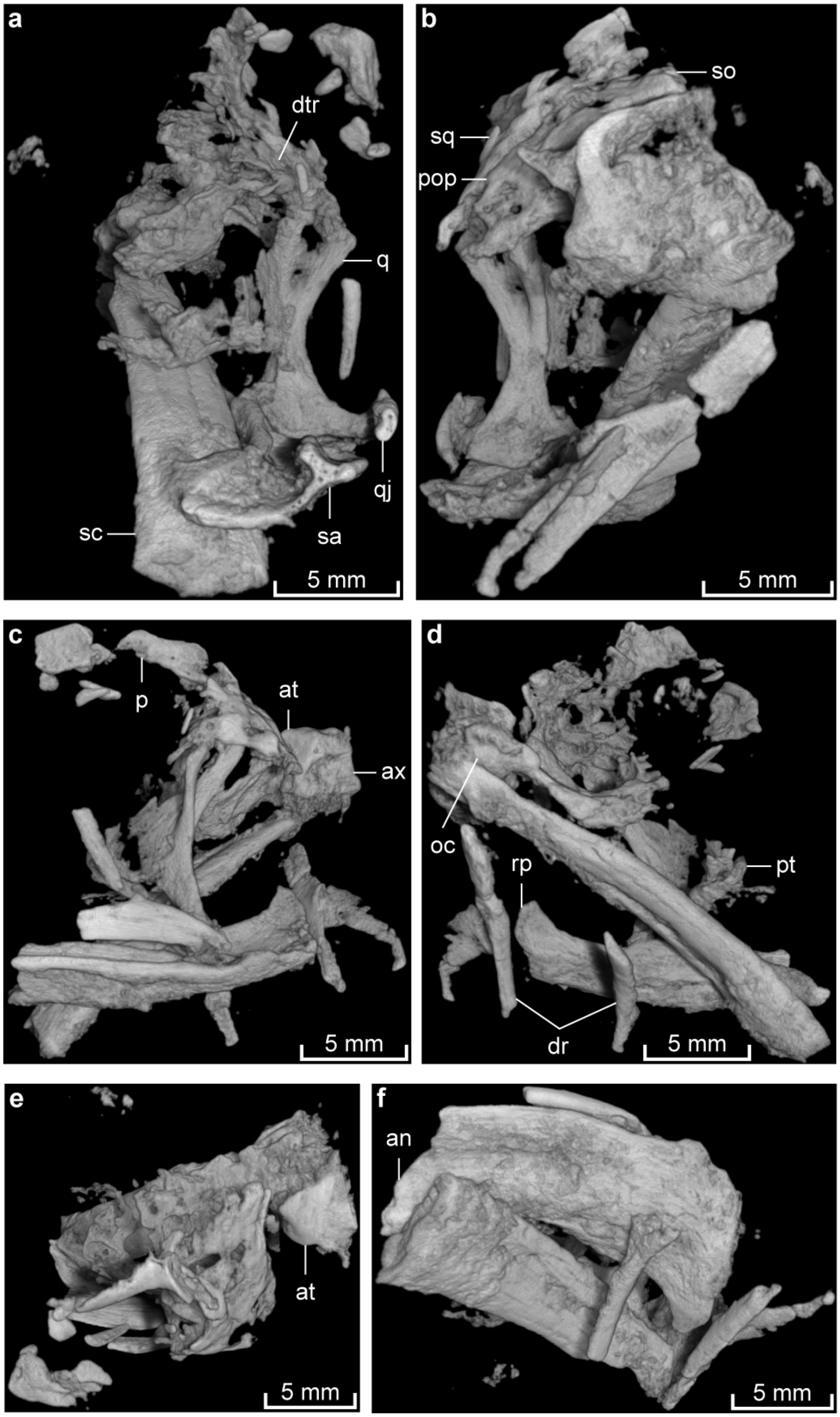
a
dtr
q
qj
sa
sc
5 mm
b
so
sq
pop
5 mm
c
p
at
ax
5 mm
d
oc
rp
pt
dr
5 mm
e
at
5 mm
f
an
5 mm

**Supplementary Figure 2. $\mu$CT rendered images of the posterior skull of *Natovenator polydontus* (MPC-D 102/114, holotype)**. Posterior skull in anterior (**a**), posterior (**b**), left lateral (**c**), right lateral (**d**), dorsal (**e**), and ventral (**f** ) views. Abbreviations: an, angular; at, atlas; ax, axis; dr, dorsal rib; dtr, dorsal tympanic recess; p, parietal; pop, paroccipital process; pt, pterygoid; q, quadrate; qj, quadratojugal; oc, occipital condyle; rp, retroarticular process; sa, surangular; sc, scapula; sq, squamosal; so, supraoccipital.

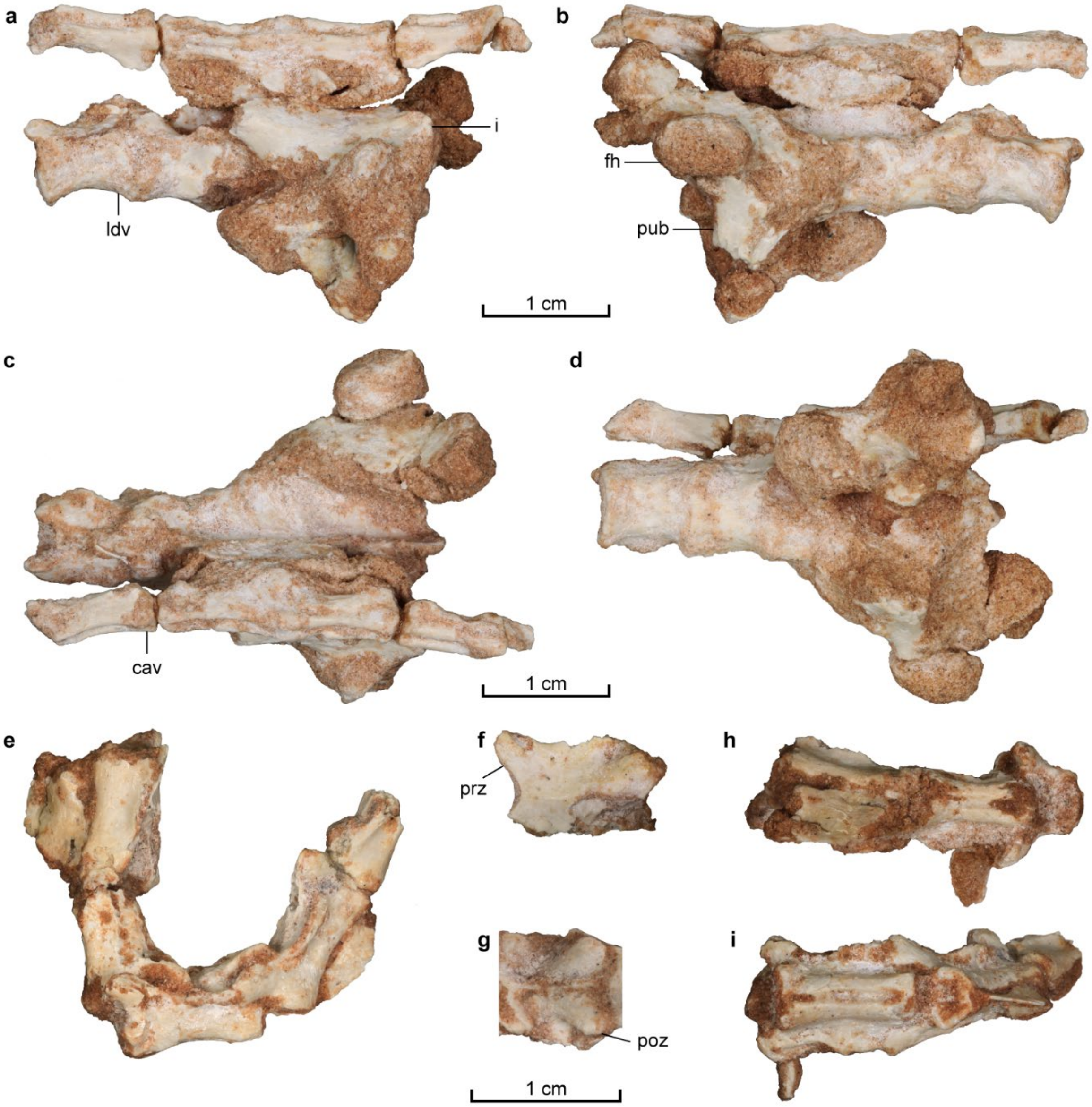


**Supplementary Figure 3. Sacrum and caudal vertebrae of *Natovenator polydontus* (MPC-D 102/114, holotype). a–d**, Sacrum with mid-caudal vertebrae on top in left lateral (**a**), right lateral (**b**), dorsal (**c**), and ventral (**d**) views. **e**, Anterior caudal vertebrae in left lateral (**e**), dorsal (**f**, **g**), and ventral (**h**, **i**) views. **f** and **h** show the anteriormost vertebrae in the preserved caudal series, whereas **g** and **i** are focused on the following ones. Abbreviations: cav, caudal vertebra; fh, femoral head; i, ilium; ldv, last dorsal vertebra; poz; postzygapophysis; prz, prezygapophysis; pub, pubis.

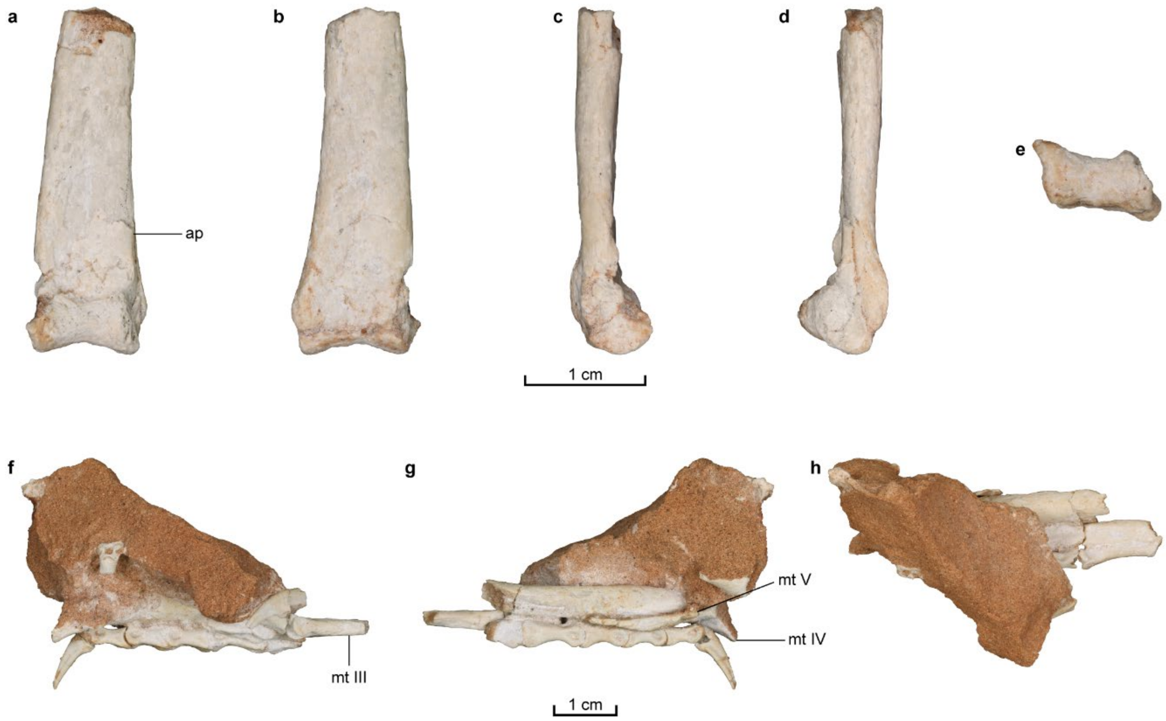


**Supplementary Figure 4. Hind limb and pedal elements of *Natovenator polydontus* (MPC-D 102/114, holotype). a**–**e**, Distal part of the left tibiotarsus in anterior (**a**), posterior (**b**), medial (**c**), lateral (**d**), and distal (**e**) views. **f**–**h**, Right foot in medial (**f**), lateral (**g**), and dorsal (**h**) views. Abbreviations: ap, ascending process of astragalus; mt III, metatarsal III; mt IV, metatarsal IV; mt V, metatarsal V.

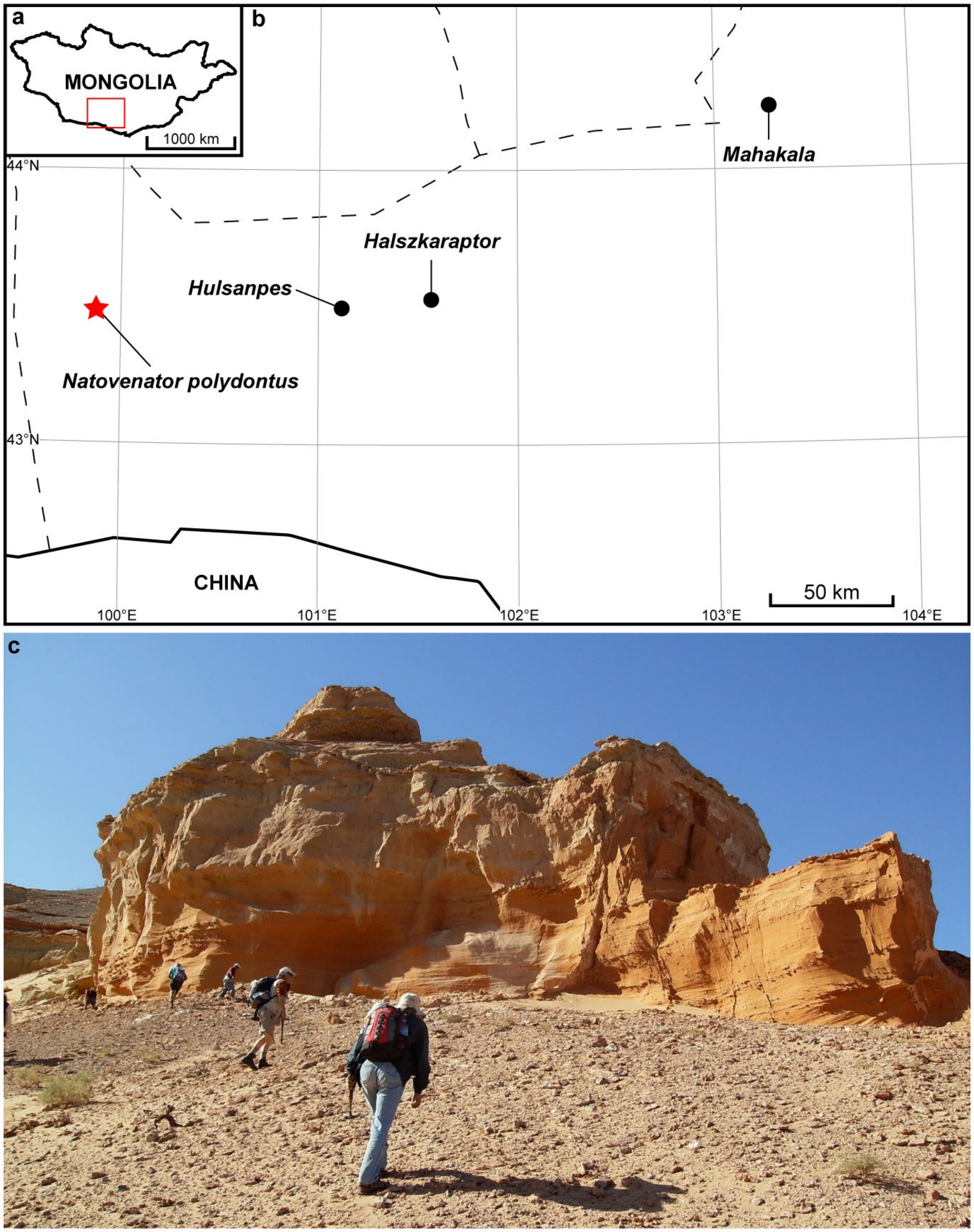


**Supplementary Figure 5. Locality of halszkaraptorines. a**, A map of Mongolia. **b**, A magnified map (red rectangle in **a**) showing occurrences of halszkaraptorines, including

*Natovenator polydontus*. **c**, The site from which *Natovenator polydontus* (MPC-D 102/114) was recovered.

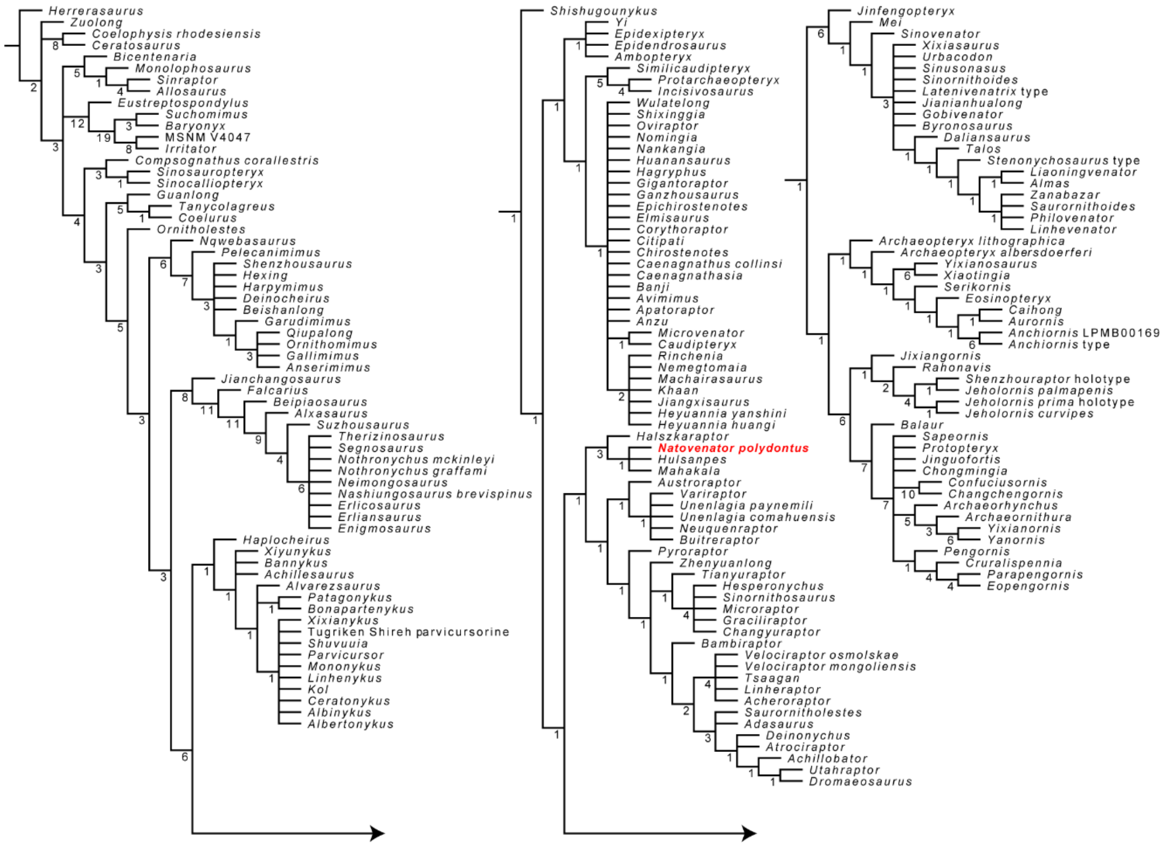


**Supplementary Figure 6. Strict consensus of the most parsimonious trees found in the phylogenetic analysis.** Numbers at each node indicate Bremer support values.

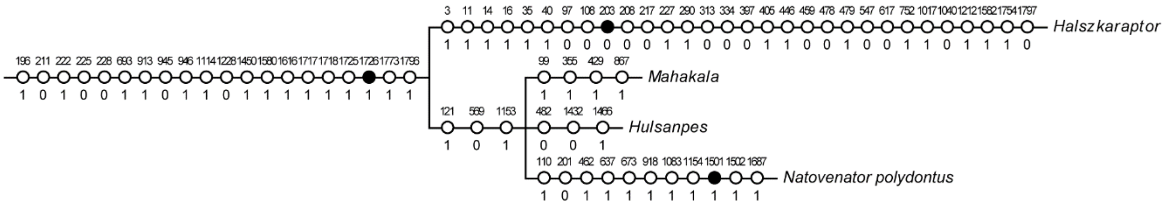


**Supplementary Figure 7. Phylogeny of Halszkaraptorinae on the strict consensus tree with synapomorphies.** Black circles indicate unambiguous synapomorphies, while white circles indicate ambiguous ones.

# 3. Supplementary Tables

Supplementary Table 1. Selected measurements of MPC-D 102/114.

| Element | Measurement (mm) |
|---|---|
| Skull length (estimated) | 66.29 |
| Premaxilla body length | 8.5 |
| Preorbital length of the skull | 35.06 |
| Orbit length (right, mid-height) | 18.92 |
| Orbit height (right, mid-height) | 16.6 |
| Antorbital fossa length (right) | 7.15 |
| Antorbital fenestra length (right) | 13.87 |
| Antorbital fenestra height (right) | 9.72 |
| External naris length (right) | 11.04 |
| Axial centrum length | 14.58 |
| 3rd cervical centrum length | 14.83 |
| 4th cervical centrum length | 15.04 |
| 5th cervical centrum length (estimated) | 15.53 |
| 6th cervical centrum length | 16.23 |
| 7th cervical centrum length | 15.78 |
| 8th cervical centrum length | 13.68 |
| 9th cervical centrum length | 12.55 |
| 10th cervical centrum length | 12.11 |
| 1st dorsal centrum length | 7.96 |
| 2nd dosal centrum length | 7.19 |
| 3rd dorsal centrum length | 7.25 |
| 4th dorsal centrum length | 7.39 |
| 5th dorsal centrum length | 7.11 |
| 6th dorsal centrum length | 7.76 |
| 7th dorsal centrum length | 7.24 |
| 8th dorsal centrum length (estimated) | 7.73 |
| 9th dorsal centrum length (estimated) | 7.35 |
| 10th dorsal centrum length | 7.78 |
| 11th dorsal centrum length | 7.57 |
| Last dorsal centrum length | 7.33 |
| 1st sacral centrum length | 7.13 |
| Metacarpal I length (left) | 7.13 |
| Metacarpal II length (left) | 17.16 |
| Metacarpal III length (left) | 18.11 |
| Manual phalanx I-1 length (left) | 11.82 |
| Manual phalanx II-1 length (left) | 8.71 |
| Pedal phalanx I-2 (ungual) length (right) | 10.32 |
| Pedal phalanx II-1 length (right) | 7.43 |
| Pedal phalanx IV-1 length (right) | 10.35 |
| Pedal phalanx IV-2 length (right) | 8.33 |
| Pedal phalanx IV-3 length (right) | 7.53 |
| Pedal phalanx IV-4 length (right) | 6.77 |
| Pedal phalanx IV-5 (ungual) length (right) | 10.63 |

Supplementary Table 2. Scan parameters of SkyScan1276.

| **Scanner** | **SkyScan1276** |
|---|---|
| Number Of Files | 1675 |
| Number Of Rows | 2688 |
| Number Of Columns | 4032 |
| Filename Index Length | 8 |
| Partial Width | OFF |
| Image crop origin X | 0 |
| Image crop origin Y | 0 |
| Camera binning | 1x1 |
| Image Rotation | 0.25000 |
| Optical Axis (line) | 1319 |
| Object to Source (mm) | 91.821 |
| Camera to Source (mm) | 159.798 |
| Source Voltage (kV) | 100 |
| Source Current (uA) | 40 |
| Image Pixel Size (um) | 10.047000 |
| Scaled Image Pixel Size (um) | 10.047000 |
| Depth (bits) | 16 |
| Reference Intensity | 58000 |
| Exposure (ms) | 3000 |
| Rotation Step (deg) | 0.215 |
| Use 360 Rotation | YES |
| Scanning position | 186.450 mm |
| Frame Averaging | ON (4) |
| Flat Field Correction | ON |
| Random Movement | OFF (20) |
| Filter | Al+Cu |
| Gantry direction | CC |
| Rotation Direction | CC |
| Intrinsic CS rotation in degree | 180.0 |
| Type of Detector Motion | STEP AND SHOOT |
| Scanning Trajectory | ROUND |
| Number of connected scans | 4 |
| Current scan number | 1 |
| Number of lines to be reconstructed | 1700 |
| Suggested HU - Calibration | 180000 |
| Number Of Horizontal Offset Positions | 1 |
| Scan duration | 5h:35m:3s |
| Maximum vertical TS | 5.0 |

Supplementary Table 3. Scan parameters of 620 Versa.

| **Scanner** | **620 Versa** |
|---|---|
| Imaging Mode | Tomography |
| Camera Binning | 2 |
| Camera Temperature | -59°C |
| File Data Type | USHORT |
| Display Data Type | FLOAT |
| Image Size | 1024 x 1024 |
| File Size | 1802.000 MB |
| Source Filter | Air |
| Source Setting | 70 kV, 124 µA |
| Source-RA distance | 32.3192 mm |
| Detector-RA distance | 70.4573 mm |
| Pixel Size | 21.5275 µm |
| Optical Magnification | 0.3944 : ”0.4X” |
| Exposure Time | 1.0000 sec |
| Camera Readout | Fast (3.0 MHz) |
| Heated Sample Temp | NA |
| Date and Time | 05/13/2020, 11:11:38.638 |
| Frames/Image | NA |
| Exp/Frame | NA |
| **Source Information** | |
| Power | 8.5 |
| Voltage | 70.01 |
| Target Current | 121.35 |
| Tube Current | 201.71 |
| Centering X | -107.18 |
| Centering Y | 77.67 |
| Vacuum Level | 0.00 |
| Focus Current | 1455.25 |
| Hours on target | 21.37 |
| Target turn # | 0 |
| Filament | 6.890 |
| Cold Cathode Status | Off |
| **Axes (Image 1 of 901)** | |
| Sample X | -2245.750 µm |
| Sample Y | -17 381.152 µm |
| Sample Z | -10 750.900 µm |
| Sample Theta | -114.993 deg |
| Source Z | 184.444 mm |
| Detector Z | -268.792 mm |
| CCD Z | -193 807.906 µm |
| CCD X | -59 014.199 µm |
| Filter Wheel | 180.000 deg |
| Source X | 0.000 µm |

## 4. Supplementary References


1. Cau, A. *et al.* Synchrotron scanning reveals amphibious ecomorphology in a new clade of bird-like dinosaurs. *Nature* **552,** 395–399 (2017).

2. Ostrom, J. H. Osteology of *Deinonychus antirrhopus*, an unusual theropod from the Lower Cretaceous of Montana. *B. Peabody Mus. Nat. Hist.* **30,** 1–165 (1969).

3. Barsbold, R. & Osmólska, H. The skull of *Velociraptor* [Theropoda] from the Late Cretaceous of Mongolia. *Acta Palaeontol. Pol.* **44,** 189–219 (1999).

4. Burnham, D. A. *et al.* Remarkable new birdlike dinosaur (Theropoda: Maniraptora) from the Upper Cretaceous of Montana. *Paleontol. Contrib.* **13,** 1–14 (2000).

5. Norell, M. A. & Makovicky, P. J. in *The Dinosauria* (eds Weishampel, D. B., Dodson, P. & Osmólska, H.) 2nd, 196–209 (University of California Press, 2004).

6. Makovicky, P. J., Apesteguia, S. & Agnolin, F. L. The earliest dromaeosaurid theropod from South America. *Nature* **437,** 1007–1011 (2005).

7. Norell, M. A. *et al.* A new dromaeosaurid theropod from Ukhaa Tolgod (Ömnögov, Mongolia). *Am. Mus. Novit.* **3545***,* 1–51 (2006).

8. Xu, X. *et al.* The taxonomic status of the Late Cretaceous dromaeosaurid *Linheraptor exquisitus* and its implications for dromaeosaurid systematics. *Vertebrat. Palasiatic.* **53,** 29–62 (2015).

9. Currie, P. J. & Evans, D. C. Cranial anatomy of new specimens of *Saurornitholestes langstoni* (Dinosauria, Theropoda, Dromaeosauridae) from the Dinosaur Park Formation (Campanian) of Alberta. *Anat. Rec.* **303,** 691–715 (2019).

10. Turner, A. H., Makovicky, P. J. & Norell, M. A. A review of dromaeosaurid systematics and paravian phylogeny. *B. Am. Mus. Nat. Hist.* **371***,* 1–206 (2012).

11. Marsh, O. C. *Odontornithes: a monograph on the extinct toothed birds of North America.* 201 (Washington D. C.: Government Printing Office, 1880).

12. Field, D. J. *et al.* Complete *Ichthyornis* skull illuminates mosaic assembly of the avian head. *Nature* **557,** 96–100 (2018).

13. Clark, J. M., Altangerel, P. & Norell, M. A. The skull of *Erlicosaurus andrewsi*, a Late Cretaceous "Segnosaur" (Theropoda: Therizinosauridae) from Mongolia. *Am. Mus. Novit.* **3115***,* 1–39 (1994).

14. Pu, H. *et al.* An unusual basal therizinosaur dinosaur with an ornithischian dental arrangement from Northeastern China. *PLOS ONE* **8,** e63423 (2013).

15. Gianechini, F. A., Makovicky, P. J. & Apesteguía, S. The cranial osteology of *Buitreraptor gonzalezorum* Makovicky, Apesteguía, and Agnolín, 2005 (Theropoda, Dromaeosauridae), from the Late Cretaceous of Patagonia, Argentina. *J. Vertebr. Paleontol.* **37,** e1255639 (2017).

16. Novas, F. E., Pol, D., Canale, J. I., Porfiri, J. D. & Calvo, J. O. A bizarre Cretaceous theropod dinosaur from Patagonia and the evolution of Gondwanan dromaeosaurids. *P. Roy. Soc. B-Biol. Sci.* **276,** 1101–1107 (2009).

17. Xu, X. & Wu, X.-C. Cranial morphology of *Sinornithosaurus millenii* Xu et al. 1999 (Dinosauria: Theropoda: Dromaeosauridae) from the Yixian Formation of Liaoning, China. *Can. J. Earth Sci.* **38,** 1739–1752 (2001).

18. Turner, A. H., Pol, D., Clarke, J. A., Erickson, G. M. & Norell, M. A. A Basal Dromaeosaurid and Size Evolution Preceding Avian Flight. *Science* **317,** 1378–1381 (2007).

19. Turner, A. H., Pol, D. & Norell, M. A. Anatomy of *Mahakala omnogovae* (Theropoda: Dromaeosauridae), T¨ogr¨ogiin Shiree, Mongolia. *Am. Mus. Novit.* **3722***,* 1–66 (2011).

20. Makovicky, P. J. & Norell, M. A. in *The Dinosauria* (eds Weishampel, D. B., Dodson, P. & Osmólska, H.) 2nd, 184–195 (University of California Press, 2004).

21. Currie, P. J. New information on the anatomy and relationships of *Dromaeosaurus albertensis* (Dinosauria: Theropoda). *J. Vertebr. Paleontol.* **15,** 576–591 (1995).

22. Xu, X. & Norell, M. A. A new troodontid dinosaur from China with avian-like sleeping posture. *Nature* **431,** 838–841 (2004).

23. Yin, Y.-L., Pei, R. & Zhou, C.-F. Cranial morphology of *Sinovenator changii* (Theropoda: Troodontidae) on the new material from the Yixian Formation of western Liaoning, China. *PeerJ* **6,** e4977 (2018).

24. Brinkman, D. L., Cifelli, R. L. & Czaplewski, N. J. First Occurrence of *Deinonychus antirrhopus* (Dinosauria: Theropoda) from the Antlers Formation (Lower Cretaceous: Aptian-Albian) of Oklahoma. *Okla. Geol. Surv. Bull.* **146,** 1–27 (1998).

25. Norell, M. A., Makovicky, P. J. & Clark, J. M. in *Feathered dragons: Studies on the transition from dinosaurs to birds* (eds Currie, P. J., Koppelhus, E. B., Shugar, M. A. & Wright, J. L.) 133–143 (Indiana University Press, 2004).

26. Sues, H.-D. & Averianov, A. Dromaeosauridae (Dinosauria: Theropoda) from the Bissekty Formation (Upper Cretaceous: Turonian) of Uzbekistan and the phylogenetic position of *Itemirus medullaris* Kurzanov, 1976. *Cretaceous Res*. **51,** 225–240 (2014).

27. Norell, M. A. *et al.* A review of the Mongolian Cretaceous dinosaur *Saurornithoides* (Troodontidae: Theropoda). *Am. Mus. Novit.* **3654**, 1–63 (2009).

28. Hwang, S. H., Norell, M. A., Qiang, J. & Keqin, G. New specimens of *Microraptor zhaoianus* (Theropoda: Dromaeosauridae) from northeastern China. *Am. Mus. Novit.* **3381***,* 1–44 (2002).

29. Xu, X. *et al.* A new dromaeosaurid (Dinosauria: Theropoda) from the Upper Cretaceous Wulansuhai Formation of Inner Mongolia, China. *Zootaxa* **2403,** 1–9 (2010).

30. Cau, A. The body plan of *Halszkaraptor escuilliei* (Dinosauria, Theropoda) is not a transitional form along the evolution of dromaeosaurid hypercarnivory. *PeerJ* **8,** e8672 (2020).

31. Gianechini, F. A., Makovicky, P. J., Apesteguía, S. & Cerda, I. Postcranial skeletal anatomy of the holotype and referred specimens of *Buitreraptor gonzalezorum* Makovicky, Apesteguía and Agnolín 2005 (Theropoda, Dromaeosauridae), from the Late Cretaceous of Patagonia. *PeerJ* **6,** e4558 (2018).

32. Norell, M. A. & Makovicky, P. J. Important features of the dromaeosaurid skeleton II: Information from newly collected specimens of *Velociraptor mongoliensis*. *Am. Mus. Novit.* **3282***,* 1–45 (1999).

33. Novas, F. E., Egli, F. B., Agnolin, F. L., Gianechini, F. A. & Cerda, I. Postcranial osteology of a new specimen of *Buitreraptor gonzalezorum* (Theropoda, Unenlagiidae). *Cretaceous Res*. **83,** 127–167 (2018).

34. Tokaryk, T. T. & Harington, C. R. *Baptornis* sp. (Aves: Hesperornithiformes) from the Judith River Formation (Campanian) of Saskatchewan, Canada. *J. Paleontol*. **66,** 1010–1012 (1992).

35. Rees, J. & Lindgren, J. Aquatic birds from the Upper Cretaceous (Lower Campanian) of Sweden and the biology and distribution of hesperornithiforms. *Palaeontology* **48,** 1321–1329 (2005).

36. Kuroda, N. Morpho-anatomical analysis of parallel evolution between Diving Petrel and Ancient Auk. *J. Yamashina Inst. Ornithol*. **5,** 111–137 (1967).

37. Brocklehurst, R. J., Schachner, E. R., Codd, J. R. & Sellers, W. I. Respiratory evolution

in archosaurs. *Philos. T. Roy. Soc. B* **375,** 20190140 (2020).

38. Motta, M. J., Egli, F. B. & Novas, F. E. Tail anatomy of *Buitreraptor gonzalezorum* (Theropoda, Unenlagiidae) and comparisons with other basal paravians. *Cretaceous Res.* **83,** 168–181 (2018).

39. Kirkland, J. I., Gaston, R. & Burge, D. A large dromaeosaur (Theropoda) from the Lower Cretaceous of eastern Utah. *Hunteria* **2,** 1–16 (1993).

40. Xu, X., Wang, X.-L. & Wu, X.-C. A dromaeosaurid dinosaur with a filamentous integument from the Yixian Formation of China. *Nature* **401,** 262–266 (1999).

41. Burnham, D. A. in *Feathered dragons: Studies on the transition from dinosaurs to birds* (eds Currie, P. J., Koppelhus, E. B., Shugar, M. A. & Wright, J. L.) 67–111 (Indiana University Press, 2004).

42. Xu, X. & Wang, X. A new dromaeosaur (Dinosauria: Theropoda) from the Early Cretaceous Yixian Formation of western Liaoning. *Vertebrat. Palasiatic.* **42,** 111–119 (2004).

43. Han, G. *et al.* A new raptorial dinosaur with exceptionally long feathering provides insights into dromaeosaurid flight performance. *Nat. Commun.* **5,** 1–9 (2014).

44. Zheng, X., Xu, X., You, H., Zhao, Q. & Dong, Z. A short-armed dromaeosaurid from the Jehol Group of China with implications for early dromaeosaurid evolution. *P. Roy. Soc. B-Biol. Sci.* **277,** 211–217 (2010).

45. Xu, X. & Qin, Z.-C. A new tiny dromaeosaurid dinosaur from the Lower Cretaceous Jehol Group of western Liaoning and niche differentiation among the Jehol dromaeosaurids. *Vertebrat. Palasiatic.* **55,** 129–144 (2017).

46. Currie, P. J. & Carabajal, A. P. A new specimen of *Austroraptor cabazai* Novas, Pol, Canale, Porfiri and Calvo, 2008 (Dinosauria, Theropoda, Unenlagiidae) from the latest Cretaceous (Maastrichtian) of Río Negro, Argentina. *Ameghiniana* **49,** 662–667 (2012).

47. Botelho, J. F. *et al.* New developmental evidence clarifies the evolution of wrist bones in the dinosaur–bird transition. *PLOS Biol.* **12,** 1–13 (2014).

48. Hutchinson, J. R. The evolution of femoral osteology and soft tissues on the line to extant birds (Neornithes). *Zool. J. Linn. Soc.* **131,** 169–197 (2001).

49. Forster, C. A., O'Connor, P. M., Chiappe, L. M. & Turner, A. H. The osteology of the Late Cretaceous paravian *Rahonavis ostromi* from Madagascar. *Palaeontol. Electronica* **23,** a29 (2020).

50. Osmólska, H. *Hulsanpes perlei* n.g. n.sp. (Deinonychosauria, Saurischia, Dinosauria) from the Upper Cretaceous Barun Goyot Formation of Mongolia. *Neues Jahrb. Geol. P., M.* **1982**, 440–448 (1982).

51. Cau, A. & Madzia, D. Redescription and affinities of *Hulsanpes perlei* (Dinosauria, Theropoda) from the Upper Cretaceous of Mongolia. *PeerJ* **6,** e4868 (2018).

52. Novas, F. E. & Pol, D. New evidence on deinonychosaurian dinosaurs from the Late Cretaceous of Patagonia. *Nature* **433,** 858–861 (2005).
