# Supplementary material for: A non-avian dinosaur with a streamlined body exhibits potential adaptations for swimming
Source: Commun Biol. 2022 Dec 1;5:1185. doi: 10.1038/s42003-022-04119-9 (PMC9715538; doi:10.1038/s42003-022-04119-9)
Supplement: Supplementary file 3 — Description of Additional Supplementary Files [file 42003_2022_4119_MOESM3_ESM.pdf]

Description of Additional Supplementary Files

File name: Supplementary Data 1
Description: : Data matrix of Natovenator polydontus used in our phylogenetic analysis.
