# Supplementary material for: A non-avian dinosaur with a streamlined body exhibits potential adaptations for swimming
Source: Commun Biol. 2022 Dec 1;5:1185. doi: 10.1038/s42003-022-04119-9 (PMC9715538; doi:10.1038/s42003-022-04119-9)
Supplement: Supplementary file 5 — Reporting Summary [file 42003_2022_4119_MOESM5_ESM.pdf]

nature portfolio

Corresponding author(s): Yuong-Nam Lee

Last updated by author(s): Oct 4, 2022

Reporting Summary

Nature Portfolio wishes to improve the reproducibility of the work that we publish. This form provides structure for consistency and transparency in reporting. For further information on Nature Portfolio policies, see our Editorial Policies and the Editorial Policy Checklist.

Statistics

For all statistical analyses, confirm that the following items are present in the figure legend, table legend, main text, or Methods section.

n/a	Confirmed	
☒	☐	The exact sample size (*n*) for each experimental group/condition, given as a discrete number and unit of measurement
☒	☐	A statement on whether measurements were taken from distinct samples or whether the same sample was measured repeatedly
☒	☐	The statistical test(s) used AND whether they are one- or two-sided<br>*Only common tests should be described solely by name; describe more complex techniques in the Methods section.*
☒	☐	A description of all covariates tested
☒	☐	A description of any assumptions or corrections, such as tests of normality and adjustment for multiple comparisons
☒	☐	A full description of the statistical parameters including central tendency (e.g. means) or other basic estimates (e.g. regression coefficient) AND variation (e.g. standard deviation) or associated estimates of uncertainty (e.g. confidence intervals)
☒	☐	For null hypothesis testing, the test statistic (e.g. *F*, *t*, *r*) with confidence intervals, effect sizes, degrees of freedom and *P* value noted<br>*Give P values as exact values whenever suitable.*
☒	☐	For Bayesian analysis, information on the choice of priors and Markov chain Monte Carlo settings
☒	☐	For hierarchical and complex designs, identification of the appropriate level for tests and full reporting of outcomes
☒	☐	Estimates of effect sizes (e.g. Cohen's *d*, Pearson's *r*), indicating how they were calculated

Our web collection on statistics for biologists contains articles on many of the points above.

Software and code

Policy information about availability of computer code

Data collection	Dragonfly from Object Research Systems for µCT (or X-ray microscope) data.<br>Adobe Photoshop CC and Illustrator CC for image production and drawings.
Data analysis	TNT v. 1.5 for phylogenetic analysis

For manuscripts utilizing custom algorithms or software that are central to the research but not yet described in published literature, software must be made available to editors and reviewers. We strongly encourage code deposition in a community repository (e.g. GitHub). See the Nature Portfolio guidelines for submitting code & software for further information.

Data

Policy information about availability of data

All manuscripts must include a data availability statement. This statement should provide the following information, where applicable:

- Accession codes, unique identifiers, or web links for publicly available datasets
- A description of any restrictions on data availability
- For clinical datasets or third party data, please ensure that the statement adheres to our policy

Data matrix of Natovenator is included in the Supplementary Data 1. Data matrix of other taxa can be found in Cau (2020). The µCT scanned images are deposited at.

Human research participants

Policy information about studies involving human research participants and Sex and Gender in Research.

Reporting on sex and gender	N/A
Population characteristics	N/A
Recruitment	N/A
Ethics oversight	N/A

Note that full information on the approval of the study protocol must also be provided in the manuscript.

Field-specific reporting

Please select the one below that is the best fit for your research. If you are not sure, read the appropriate sections before making your selection.

☐ Life sciences ☐ Behavioural & social sciences ☒ Ecological, evolutionary & environmental sciences

For a reference copy of the document with all sections, see nature.com/documents/nr-reporting-summary-flat.pdf

Ecological, evolutionary & environmental sciences study design

All studies must disclose on these points even when the disclosure is negative.

Study description	We describe a new taxon of a theropod dinosaur and examine its phylogenetic position and ecology.
Research sample	The specimen (MPC-D 102/114) described here was collected from a field work in 2008.
Sampling strategy	The specimen (MPC-D 102/114) was the sole sample available.
Data collection	Sungjin Lee did scanning, measurement, and character coding. Scanning parameters are included in Supplementary Information. Measurements and character coding were done by examining the specimen (MPC-D 102/114). Rib angle data of other taxa were collected from 3D images of skeletons for extant birds and photographs of a fossil specimen in Turner et al. (2021) for Shri devi.
Timing and spatial scale	The specimen (MPC-D 102/114) was collected from Hermiin Tsav of the Gobi Desert, Mongolia in 2008 during the Korea-Mongolia International Dinosaur Expedition.
Data exclusions	As mentioned in the Methods section, four taxa (Alnashetri, Shanag, Fukuivenator, and Hesperornithoides) were excluded from our phylogenetic analysis to prevent collapses of major clades.
Reproducibility	The Methods section describes how the phylogenetic analysis was done.
Randomization	N/A
Blinding	N/A

Did the study involve field work? ☒ Yes ☐ No

Field work, collection and transport

Field conditions	Desert climate
Location	Hermiin Tsav, Omnogovi Province, Mongolia
Access & import/export	Institute of Paleontology in Mongolia, with permission from the Mongolian government, provided access to the location and specimen.
Disturbance	No disturbance

Reporting for specific materials, systems and methods

We require information from authors about some types of materials, experimental systems and methods used in many studies. Here, indicate whether each material, system or method listed is relevant to your study. If you are not sure if a list item applies to your research, read the appropriate section before selecting a response.

Materials & experimental systems

n/a	Involved in the study
☒	☐ Antibodies
☒	☐ Eukaryotic cell lines
☐	☒ Palaeontology and archaeology
☒	☐ Animals and other organisms
☒	☐ Clinical data
☒	☐ Dual use research of concern

Methods

n/a	Involved in the study
☒	☐ ChIP-seq
☒	☐ Flow cytometry
☒	☐ MRI-based neuroimaging

Palaeontology and Archaeology

Specimen provenance	Hermiin Tsav, Omnogovi Province, Mongolia
Specimen deposition	Institute of Paleontology, Mongolian Academy of Sciences, Ulaanbaatar, Mongolia
Dating methods	No additional dating procedure was taken.

☒ Tick this box to confirm that the raw and calibrated dates are available in the paper or in Supplementary Information.

Ethics oversight	Seoul National University, Seoul, Korea

Note that full information on the approval of the study protocol must also be provided in the manuscript.
